# The Key Role of NAD^+^ in Anti-Tumor Immune Response: An Update

**DOI:** 10.3389/fimmu.2021.658263

**Published:** 2021-04-15

**Authors:** Fabio Morandi, Alberto Leonardo Horenstein, Fabio Malavasi

**Affiliations:** ^1^ Laboratorio Cellule Staminali Post-Natali e Terapie Cellulari, IRCCS Istituto Giannina Gaslini, Genoa, Italy; ^2^ Dipartimento Scienze Mediche, Università di Torino, Centro Ricerche Medicina Sperimentale (CeRMS) and Fondazione Ricerca Molinette Onlus, Torino, Italy

**Keywords:** NAD^+^, human tumors, immune response, CD38, T cells

## Abstract

Nicotinamide adenine dinucleotide (NAD^+^) is an important molecule that functions as a co-enzyme in numerous metabolic processes. Generated both through *de novo* synthesis and *via* salvage pathways, NAD^+^ is the substrate for a variety of NAD^+^-consuming enzymes. Among them is CD38, a cell surface ecto-enzyme widely expressed on different types of cells and endowed with the function of cADP-ribose synthases/NAD^+^ glycohydrolase. Surface CD38 expression is increased in different hematological and solid tumors, where it cooperates with other ecto-enzymes to produce the immunosuppressive molecule adenosine (ADO). Few studies have explored the correlation of NAD^+^ levels with T-cell mediated anti-tumor response in preclinical models. We therefore discuss these novel findings, examining the possible contribution of NAD^+^ depletion, along with ADO production, in the immunosuppressive activities of CD38 in the context of human tumors. Lastly, we discuss the use of pharmacological inhibitors of CD38 and supplementation of different NAD^+^ precursors to increase NAD^+^ levels and to boost T cell responses. Such molecules may be employed as adjuvant therapies, in combination with standard treatments, for cancer patients.

## Introduction

Nicotinamide adenine dinucleotide (NAD^+^) and its reduced/phosphorylated forms (NADH, NADP^+^ and NADPH) are key molecules in cellular metabolism and energy production, acting as hybrid-accepting and hybrid-donating co-enzymes in different biological reactions. NAD^+^ and NADH are then inter-converted by hybrid transfer and not consumed (1). NAD^+^ can be generated *de novo* starting from tryptophan, which is converted to N-formylkynurenine by indoleamine dioxygenase or tryptophan dioxygenase. Other enzymes are involved in converting N-formylkynurenine to nicotinic acid mononucleotide (NaMN), which is adenylated by adenyl-transferases to generate nicotinic acid adenine dinucleotide (NaAD), finally converted to NAD^+^ by NAD^+^ synthetase (1). NAD^+^ can also be obtained through different salvage pathways, starting from nicotinic acid (Na) which is converted to NaMN by Na phosphoribosyltransferase (Naprt) or starting from nicotinamide (Nam) and nicotinamide riboside (NR). The latter are converted to nicotinamide mononucleotide (NMN) by Nam phosphoribosyltransferase (Nampt or PBEF) and nicotinamide riboside kinases (NamRK), respectively. NMN is finally converted to NAD^+^ by adenyl-transferases (1). In contrast with metabolic reactions, NAD^+^ is consumed during its conversion to Nam by different enzymes involved in process related to gene expression, Ca2^+^ mobilization, cell death and aging (1). These enzymes are defined as NAD^+^-dependent ADP-ribosyl transferase, and include i) ADP-ribose transferases or poly(ADP-ribose) polymerases, (ii) cADP-ribose synthases and (iii) sirtuins (NAD^+^-dependent protein deacetylases) (1).

## Inhibition of Anti-Tumor Immunity

### The Role of cADP-ribose Synthases/NAD^+^glycohydrolase

CD38 and its homologue CD157 belong to the family of cADP-ribose synthases/NAD^+^ glycohydrolases. CD38 is also part of an alternative ecto-enzymatic pathway, which involves CD203a/ENPP1 and CD73, to produce adenosine (ADO) and inosine (INO) (2, 3). CD38 expression and its role in the inhibition of anti-tumor immune response has been described in solid (4) and hematological (5–8) tumors. CD38 expression is increased during inflammation and tumor transformation, and is paralleled by CD73 up-regulation (9, 10). In chronic lymphocytic leukemia (CLL), increased CD38 expression is associated with unfavorable prognosis (11), along with T cell inhibition (12). Taken together, these observations suggest that increased CD38 expression is directly related to anti-tumor immune response suppression and/or inhibition of migration (13). Indeed, CD38^hi^ CLL cells have high NAD^+^ glycohydrolase enzymatic activity that leads to ADO production (11) (through the concerted activity of CD203a/ENPP1 and CD73) and, more importantly, to extracellular NAD^+^ consumption.

The role of CD38 in tumor-mediated inhibition of T cell functions has been extensively studied in the multiple myeloma (MM) model, where malignant plasma cells (PC) grow in a hypoxic bone marrow (BM) niche (6, 14). The niche contains a purinome, represented by different molecules interacting with extracellular nucleotides, including channels, transporters, catabolizing enzymes, intermediate products and receptors. As a result of the activity of this complex network, ADO is produced by canonical and alternative enzymatic pathways, starting from ATP or NAD^+^, respectively (3, 14). In the canonical pathway, CD39 converts extracellular ATP to AMP, whereas in the alternative pathway CD38 converts β-NAD^+^ to ADPR and Nam and CD203a/ENPP1 converts ADPR to AMP. Finally, both pathways converge to CD73, which de-phosphorylates AMP to ADO (2). The expression of ectoenzymes belonging to alternative pathway is discontinuous in the MM niche, since PC express CD38, whereas CD203a and CD73 are detectable at very low expression (15). However, the latter molecules are highly expressed by osteoblasts, osteoclasts and BM stromal cells, thus confirming the existence of the complete pathway within BM niche (6, 14). ADO exerts immunosuppressive functions, by i) inhibiting tumor cell lysis by T and NK cells, ii) inducing M2 macrophages and tolerogenic dendritic cells (DC) and iii) inducing Treg expansion (6). In this context, NAD^+^ consumption in the BM niche may account for additional immunosuppressive feature of CD38, since the alternative pathway is more active than the canonical counterpart in hypoxic conditions and at a low pH (14). Accordingly, therapeutic anti-CD38 mAbs Daratumumab and Isatuximab are able to modulate the NAD^+^ glycohydrolase enzymatic activity of CD38, likely affecting ADO production on the one hand, and NAD^+^consumption on the other (6, 16). The degree of modulation is significantly different, Isatuximab scoring the highest level (15, 17).

We elsewhere described the expression and function of ADO generation pathways in primary melanoma cells (18). In these tumor cells, CD38 and CD73 expression was constantly detected, whereas CD39 and CD203a were highly expressed in some primary melanoma cells, and low to absent in the others. Accordingly, ADO production was different, depending on the expression of each ectoenzyme (18). Melanoma cells inhibited T cell proliferation, and such inhibition was only partially reverted using inhibitors of ADO receptors: this suggests that other mechanisms may be involved. In this regard, T cell proliferation was restored by using kuromanin, a specific CD38 inhibitor, providing indirect evidence for the central role played by CD38 in melanoma-mediated immunosuppression (18). We hypothesize that NAD^+^ consumption may represent an additional immunosuppressive mechanism in *in vitro* experiments and may also represent a strategy enacted by melanoma cells *in vivo* in the tumor microenvironment to inhibit anti-tumor immune response. In this context, inoculation of Nam in a mouse model of melanoma significantly increased anti-tumor immune response through the induction of IFN-γ secretion (19). In addition, in preclinical models of melanoma, mice receiving Nam displayed a higher T cell infiltration within the tumor than those receiving placebo (20). Taken together, these studies support the role of NAD^+^ as an essential cofactor for anti-tumor T cell response in melanoma.

**Figure 1:**
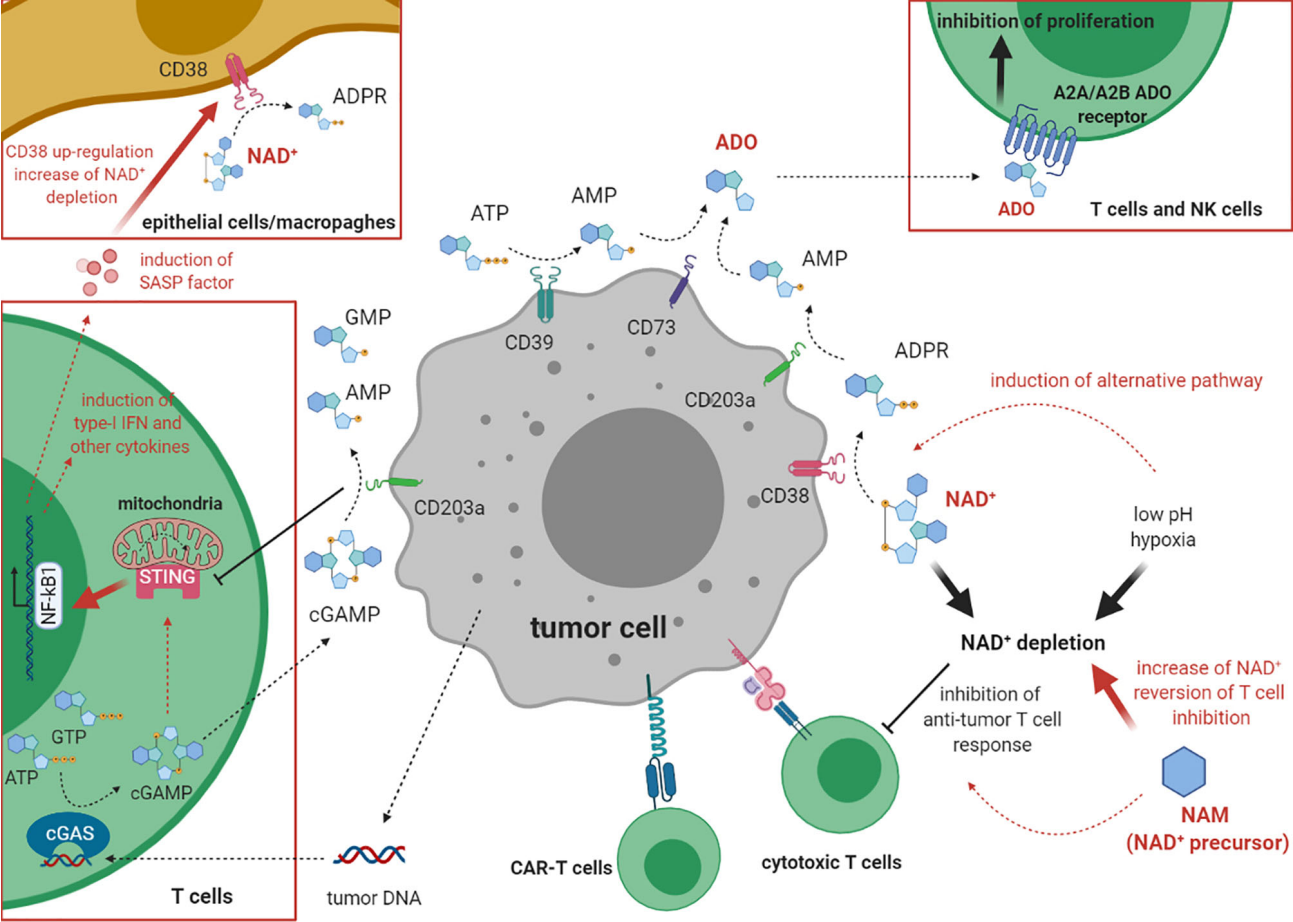
The role of cADP ribose synthase and cGAS/STING pathway in the inhibition of anti-tumor immune response. This cartoon describes the mechanism(s) underlying the inhibition of the immune response against tumor cells by CD38. On one hand, CD38 co-operates with other ectoenzymes (CD203a/ENPP1, CD73, CD39) in the production of the immunosuppressive molecule ADO. On the other hand, CD38 depletes NAD^+^ from the extracellular environment, especially in conditions with low pH and hypoxia, thus inhibiting tumor cell lysis. cGAS (on the left) metabolizes 2’,3’cyclic GMP-AMP (cGAMP) dinucleotide also acting as modulator of the immune system. Hydrolysis of cGAMP by CD203a/ENPP1 attenuates cGAS-STING signaling and the cleavage of NAD^+^.

Recent studies showed that some purinergic ectoenzymes metabolize cyclic dinucleotides, such as 2’,3’cyclic GMP-AMP (cGAMP) (21). CD203a/ENPP1 metabolizes cGAMP generating AMP and GMP (22). cGAMP is an activator of the stimulator of interferon genes (STING), which stimulate innate immunity. In brief, cyclic GMP-AMP synthase (cGAS) senses DNA released from tumors and catalyzes the conversion of GTP and ATP to cGAMP, which subsequently activates STING to activate the transcription of type 1 IFN and other cytokines, and by activating DC (21). Links between the cGAS-STING pathway with CD203a/ENPP1 and NAD^+^ have recently emerged whereby the hydrolysis of cGAMP by CD203a/ENPP1 attenuates cGAS-STING signaling and NAD^+^ cleavage (23, 24). Indeed, bacterial STING, containing a NADase-TIR domain, recognizes cyclic dinucleotides in a conformation similar to cGAMP in complex with human STING, allowing for β-NAD^+^ hydrolysis (22). NAD^+^ cleavage activity of bacterial TIR domains is conserved in the mammalian SARM1 (sterile alpha and TIR motif containing 1) NAD^+^-glycohydrolase (23, 25). In mice, high cGAMP correlated with high anti-tumor activity, by a direct triggering of the STING-dependent pathway. Consequently, inhibitors of CD203a/ENPP1 (26) could have anti-tumor activity by inhibiting the conversion of extracellular β-NAD^+^ to ADO and by inhibiting cGAMP degradation and, therefore, the secretion of SASP (senescent-associated secretory phenotype) factors which were found to increase CD38 expression (27, 28). All these findings are schematized in **Figure 1**.

### The Direct Role of NAD^+^

Recently, several reports investigated whether NAD^+^ might play a direct role in modulating anti-tumor immune response. In particular, extracellular levels of NAD^+^ are relevant for T cell functions. A study compared the metabolic activity of a hybrid T cell population with characteristics of both Th1 and Th17 cells, referred to as Th1/17 cells, to Th1 and Th17 cells (29). Indeed, Th1/17 cells display a unique metabolic phenotype, with high glutaminolysis and medium levels of glycolysis. The study showed that Th1/17 cells expressed 34 times more intracellular NAD^+^ levels than Th17 cells, due to glutaminolysis. In addition, NAD^+^ depletion, obtained through pharmacological inhibition of Nampt using FK866, downregulated IFN-γ/IL-17^+^ cells in Th1/17 cells. More importantly such cells, adoptively transferred to tumor-bearing mice, failed to control tumor growth unlike untreated cells, thus demonstrating that NAD^+^ is essential for anti-tumor activity of Th1/17 cells (29). Sirt1, a NAD^+^-dependent protein deacetylase, is a key factor, being higher in Th1/17 cells than in other subsets. Moreover, cells treated with Sirt1 inhibitors and Sirt-1-KO T cells displayed fewer IL-17^+^ cells. Accordingly, tyrosinase-reactive hybrid Th1/17 cells differentiated *ex vivo* in the presence of Ex527 (Sirt1 inhibitor) displayed a lower anti-tumor activity than untreated cells in a murine melanoma model. Similar results were obtained using Sirt^fl/fl^CD4^Cre^ mouse splenic T cells retrovirally transduced with TRP-1 TCR and programmed to the hybrid Th1/17 phenotype. These findings confirmed that Sirt1 deacetylase activity is required for Th1/17 cells anti-tumor functions (29). While investigating the target of Sirt1 activity, the researchers found a reduced acetylation status in Th1/17 cells, which is partially reverted when NAD^+^ levels are decreased using pharmacological inhibitors. In particular, they analyzed *Foxo1*, a transcription factor involved in the responses of memory T cells, which is regulated by phosphorylation and acetylation. *Foxo1* phosphorylation, which correlated with its internalization and degradation, was found to be the highest in Th17 cells, and at medium levels in Th1/Th17 cells. In contrast, *Foxo1* acetylation, which attenuates its function, is reduced in both Th1 and Th1/17 cells. Thus, the transcriptional activity of *Foxo1* is the highest in Th1/17 cells due to phosphorylation/acetylation balance, which leads to higher levels of the *Foxo1* target genes *Klf2* and *Ccr7* in Th1/17 cells than in Th17 and Th1 cells. This implies an increased Th1/17 cells homing to the lung, liver, spleen, and lymph nodes after adoptive transfer. Lastly, pharmacological inhibition of NAD^+^ and Sirt1 leads to direct inhibition of *Klf2* and *Ccr7*, thus confirming that the NAD^+^-Sirt1 axis regulates differentiation, migration, effector functions and the anti-tumor response of Th1/17 cells (29).

The role of NAD^+^ in the metabolic reprogramming of T cells and in T-cell mediated anti-tumor immune response was further demonstrated using CD38^KO^ cells. Such cells lack CD38 NAD-ase activity, and consequently have higher levels of NAD^+^ and Sirt1 activity. Moreover, they display enhanced expression of the glutaminolysis pathway and of the mitochondrial biogenesis regulator PGC1a, thus confirming a metabolic phenotype similar to that of Th1/17 cells. More importantly, they controlled tumor growth in animal models more efficiently than wild-type cells, with an increased cytokine production within the tumor microenvironment. These observations suggested that CD38 loss, and consequent extracellular NAD^+^ increase, is the key to implement anti-tumor activity of T cells *in vivo*. This conclusion was reinforced by additional studies were T cells were treated with TGF-β (which mimics tumor immune suppression) and then with anti-CD38 antibody (29). Such cells were lower in CD38 expression and higher in cytokine production and Sirt1 activity than untreated cells. Accordingly, tumor-bearing mice undergoing adoptive transfer of T cells after anti-CD38 treatment had longer survival times and better control of tumor growth than mice treated with anti-CD38 or T cells alone (29).

The role of Sirt2 in the differentiation of effector memory T cells (TEM) was analyzed in breast cancer patients (30). Patients express lower levels of Sirt2 in T lymphocytes than the controls, and patients with high levels of Sirt2 exhibited a higher percentage of TEM than patients with low Sirt2 expression. The percentage of TEM was highest in patients with the worst prognosis, since such cells differentiate from naïve T cells and are mobilized to exert an anti-tumor response. The role of Sirt2 was unambiguously demonstrated in Sirt2^-/-^ mice, where naïve T cells were more abundant than TEM. Sirt2 acted through the GSK-3β deacetylation (30). This study confirmed that Sirt2, a NAD^+^ dependent deacetylase, is pivotal in the differentiation of naïve T cells to TEM. Thus, high levels of NAD^+^ in the tumor microenvironment are required for Sirt2 activation and, in turn, for T cell differentiation and for achieving powerful anti-tumor immune response. In contrast, NAD^+^ depletion, as observed in different human tumors, may lead to immune suppression.

A novel study analyzed the role of NAD^+^ in the activation of the anti-tumor T cell response (31). Genetic and metabolic analysis of the Jurkat T-ALL cell line allowed the genes involved in T cell activation to be identified. Nampt was proved as a key factor for T cell activation in both analysis, and three compounds known to target Nampt (FK866, STF-118804, and GMX1178) as the most disruptive to T cell activation (31). Accordingly, FK866 was able to inhibit T cell activation, as witnessed by the downregulation of CD69, CD25 and ICOS activation markers, inhibition of calcium flux and phosphorylation of signaling proteins. Interestingly, NAD^+^ intracellular levels were restored by adding NAD^+^ to the culture medium, thus suggesting that T cells are able to uptake NAD^+^ from the environment. This was able to restore T cell activation, confirming that high NAD^+^ levels are essential for T cell activation (31). Appealing results were obtained by comparing the NAD^+^ levels of tumor infiltrating lymphocytes (TIL) and peripheral blood lymphocytes (PBL), in samples from ovarian cancer patients and from melanoma preclinical models. NAD^+^ levels were significantly lower in TIL than in PBL, thus suggesting that the tumor microenvironment induced NAD^+^ depletion in infiltrating lymphocytes. Accordingly, the authors observed an increased nicotinate and nicotinamide metabolism KEGG pathway in TIL as compared to PBL (31). The salvage NAD^+^ synthesis pathway represents the principal source of NAD^+^ in T cells, since *Nampt* knockdown induced a decrease of about 50% of intracellular NAD^+^ levels (31). Pathways regulated by NAD^+^ in T cells were also analyzed, revealing that inhibition of NAD^+^ synthesis by FK866 altered the levels of metabolites belonging to the glutaminolysis, glycolysis and citric acid cycle pathways, thus affecting mitochondrial oxidative phosphorylation, both in Jurkat cells and in PBL. Also here it was confirmed significant decreases in lactate, citric acid, succinic acid and oxoglutaric acid, along with an increase in glucose and glutamine. As a final result, NAD^+^ deprivation reduced the number of mitochondria and their respiratory capacity, leading to lower ATP levels in T cells (31). Notably, this study analyzed the role of NAD^+^ in CAR-T cell functions against solid tumors. It was confirmed that NAD^+^ depletion by FK866 decreased *ex vivo* cytotoxic potential of CD19-41BB CAR T cells, through the downregulation of Granzyme B, IL-2 and IFN-γ secretion. In contrast, increased NAD^+^ intracellular levels (achieved by Nampt overexpression) enhanced tumor cell killing by CAR T cells (31). In line are the results obtained *in vivo* using mice subcutaneously inoculated with K562-CD19 tumor cells and then treated with CD19-41BB CAR T cells. A cohort of mice underwent intraperitoneal injection of Nam, which increased NAD^+^ intracellular levels in CAR T cells, about as much as NAD^+^ itself. This cohort showed an increased number of tumor-free mice, prolonged survival rates and a higher percentage of lymphocytes infiltrating the tumor, as compared to mice treated with CAR T cells alone. A reasonable conclusion is that supplementation with NAD^+^ precursors may increase tumor killing and CAR T cell therapeutic functions (31). Furthermore, Nam supplementation may increase the success of therapeutic strategies based on the blockade of immune checkpoints (ICB). Mice inoculated with B16F10 and MC38 tumor cell lines were then treated with anti-PD1 and anti-CTLA4, respectively, in the presence (or absence) of Nam supplementation. In both cases, the therapeutic effect of ICB was increased in mice inoculated with Nam, showing limited tumor growth and longer survival. Infiltration of T lymphocytes in the tumor was also increased, thus indicating that Nam was able to activate the anti-tumor T cell response (31). Similar results have been obtained in a mouse model of melanoma (19), where Nam administered alone as a therapeutic agent reduced tumor growth and prolonged the survival of mice. Higher levels of IFN-γ-producing cells were found in the peripheral blood of mice treated with Nam than in untreated mice. In addition, different cytokines/chemokines were modulated in Nam-treated mice (but not in untreated mice), including an increase of IL-5 and Eotaxin and a decrease of IL-10, IL-12, IL-3 and RANTES. Thus, Nam treatment is able to increase IFN-γ secretion, which is pivotal for the anti-tumor immune response, and to modulate the balance of cytokine/chemokine in the periphery (19). These studies confirmed the role of NAD^+^ in the activation of anti-tumor T cell response and clarified one of the mechanisms underlying T cell inactivation after NAD^+^ depletion, centered on metabolic reprogramming. More importantly, NAD^+^ and its precursors have been proposed as an adjuvant therapeutic strategy to improve the clinical outcome of adoptive T cell therapy in patients with solid tumors.

**Figure 2:**
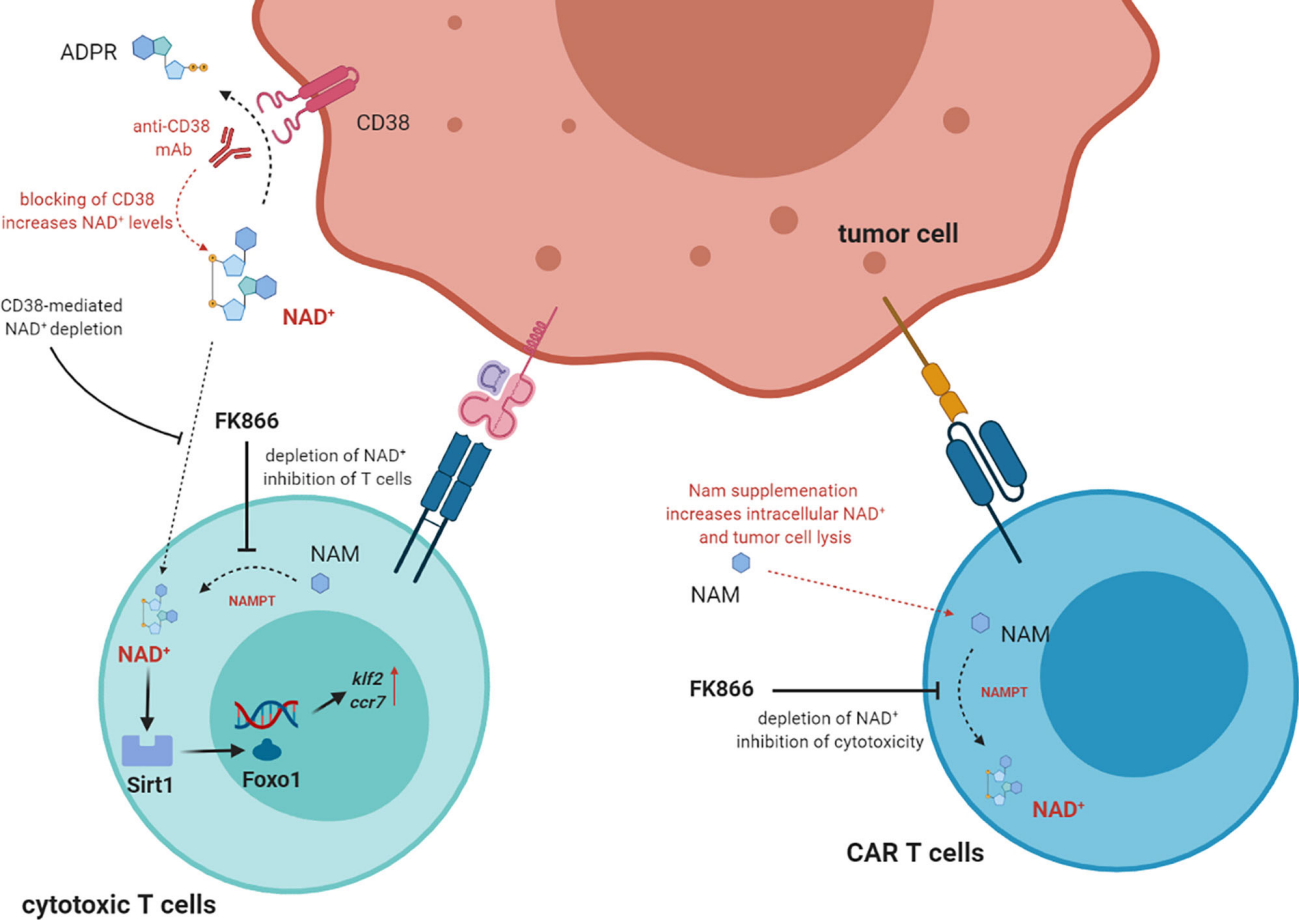
The direct role of NAD^+^ in T cell function. In T cells NAD^+^ intracellular levels are increased by NAMPT (nicotinamide phosphoribosyltransferase) which converts NAM to NAD^+^. High intracellular levels of NAD^+^ correlated with high activity of Sirt1 deacetyase activity, which in turn activate *Foxo1*and increase the expression of downstream genes *Klf2* and *Ccr7*. These effects correlated to the increase of T cell functions, including tumor cell lysis. NAD^+^ can be uptake from extracellular space, where it may be depleted by CD38 enzymatic activity. Thus, mAbs targeting CD38 may block NAD^+^depletion and increase T cell functions. Supplementation of NAM may also augment NAD^+^ levels, thus increasing tumor cell lysis. On the other hand, NAMPT inhibitors block the conversion of NAM to NAD^+^, thus inhibiting T cell functions.

The effects of NAD^+^ deprivation was analyzed on different T cell subsets and on different types of T cell response (32). Nampt serum levels were higher in transplanted patients with acute severe GvHD than in those with no signs of GvHD. Furthermore, Nampt was predominantly expressed by T lymphocytes in colonic sections of patients, and the same results were observed in mice receiving allogenic BM transplantation. Furthermore, mice receiving allogeneic BMT and treated with FK866 were clinically protected against GvHD, as witnessed by a decreased weight loss, reduced clinical GvHD scores, normal colon length and reduced histological severity scores in colon and liver (32). Accordingly, mice treated with FK866 showed fewer donor T cells and more Tregs in spleen and liver than untreated mice. Worth noting, memory T cells and other immune cell populations, including monocytes, macrophages, DC and NK cells, were not affected by FK866 treatment. Such treatment induced apoptosis in donor-derived T cells in spleen and liver. In addition, no effects were observed in mice receiving syngenic BMT, thus suggesting that inhibition of Nampt only affects alloreactive T cells (32). Upon TCR stimulation, Nampt mRNA was upregulated in effector cells but not in Tregs: these effects were paralleled by increased intracellular NAD^+^ levels. In line with this observation, FK866 treatment reduced viability and downregulated IFN-γ and TNF-α production only in effector T cells, mediated by NAD^+^ depletion. In contrast, FK866 significantly up-regulated FoxP3 expression in Tregs, through the inhibition of Sirt1-mediated acetylation, thus increasing their regulatory functions (32). Lastly, the authors setup a graft-versus-leukemia (GvL) experimental model, where mice underwent syngenic or allogenic BMT and then inoculated with leukemia or lymphoma cell lines. Mice receiving syngenic BMT rapidly developed metastatic tumors, and tumor spread was abrogated with FK866 treatment, thus suggesting a direct anti-tumor effect of this molecule. In contrast, mice receiving allogeneic BMT display a limited tumor burden (due to GvL) and a severe GvHD. In these mice, FK866 treatment reduced GvHD and suppressed tumor growth. The study demonstrated that FK866 abrogated tumor cell proliferation by depleting intracellular NAD^+^ (32). Thus, NAD^+^ is necessary for T cell proliferation and T cell responses. Targeting NAD^+^ salvage pathway may be useful for the treatment of GvHD in transplanted patients, since Nampt activity and NAD^+^ levels are the highest in allogeneic T cells, without affecting GvL and anti-tumor activity. **Figure 2** describes the role of NAD^+^ in anti-tumor T cell responses.

## Discussion

This mini review reports on recent evidence highlighting the role of NAD^+^ as a key factor in the anti-tumor T cell response. To date, a handful of studies indicate that NAD^+^ administration may boost the anti-tumor activity of canonical and engineered T cells in preclinical models of human tumors. NAD^+^ depletion brought about by immunosuppressive mechanisms, which involve NAD^+^-consuming enzymes, may dampen T cell functions. Based on these findings, it appears that CD38 immunosuppressive activity in human solid and hematological tumors, which hinges upon the production of the inhibitory molecule ADO, might exploit NAD^+^ consumption as well. This feature is more relevant in the specific microenvironment of the BM niche in MM, which is characterized by hypoxia and low pH, thus rendering the alternative pathway (which consumes NAD^+^) more effective than the canonical one. In conclusion, NAD^+^ balance must be taken into account in the treatment of cancer patients, and enzymes involved in NAD^+^ synthesis and catabolism may represent novel targets for adjuvant therapies, contributing to the success of adoptive T cell therapies. Indeed, recent studies have addressed the efficacy of CD38 inhibitors (i.e. kuromanin) in preclinical models of CLL (33, 34) in combination with standard therapies. In addition, Nam has been administered in combination with radiotherapy or chemotherapy to patients with different solid tumors, obtaining promising effects (35). Thus, pharmacological interventions aimed at restoring NAD^+^ levels may increase the efficacy of standard therapies for patients with solid and hematological tumors. In this line, other NAD^+^ precursors, such as NR and NMN, have been recently proposed to revert NAD^+^ depletion in multiple organ fibrosis (36) and aging (37), respectively. Finally, five clinical studies using Nam as adjuvant therapy are currently ongoing for cancer patients (www.clinicaltrials.gov).

## Author Contributions

FMo wrote the manuscript, AH and FMa critically revised the original draft. All authors contributed to the article and approved the submitted version.

## Funding

This work was supported by Ministero della Salute, Progetti di Ricerca Corrente and “CD38 Project”, Fondazione Ricerca Molinette, Torino, Italy.

## Conflict of Interest

The authors declare that the research was conducted in the absence of any commercial or financial relationships that could be construed as a potential conflict of interest.

The reviewer SD declared a shared affiliation with the authors AH and FMa to the handling Editor.
